# Structural and Biochemical Analysis of a Single Amino-Acid Mutant of WzzB_SF_ That Alters Lipopolysaccharide O-Antigen Chain Length in *Shigella flexneri*

**DOI:** 10.1371/journal.pone.0138266

**Published:** 2015-09-17

**Authors:** Chiung-Wen Chang, Elizabeth N. H. Tran, Daniel J. Ericsson, Lachlan W. Casey, Thierry Lonhienne, Friederike Benning, Renato Morona, Bostjan Kobe

**Affiliations:** 1 School of Chemistry and Molecular Biosciences, University of Queensland, Brisbane Qld 4072, Australia; 2 Institute for Molecular Bioscience, University of Queensland, Brisbane Qld 4072, Australia; 3 Australian Infectious Disease Research Centre, University of Queensland, Brisbane Qld 4072, Australia; 4 School of Biological Sciences, Department of Molecular and Cellular Biology, University of Adelaide, Adelaide 5005, Australia; University of Canterbury, NEW ZEALAND

## Abstract

Lipopolysaccharide (LPS), a surface polymer of Gram-negative bacteria, helps bacteria survive in different environments and acts as a virulence determinant of host infection. The O-antigen (Oag) component of LPS exhibits a modal chain-length distribution that is controlled by polysaccharide co-polymerases (PCPs). The molecular basis of the regulation of Oag chain-lengths remains unclear, despite extensive mutagenesis and structural studies of PCPs from *Escherichia coli* and *Shigella*. Here, we identified a single mutation (A107P) of the *Shigella flexneri* WzzB_SF_, by a random mutagenesis approach, that causes a shortened Oag chain-length distribution in bacteria. We determined the crystal structures of the periplasmic domains of wild-type WzzB_SF_ and the A107P mutant. Both structures form a highly similar open trimeric assembly in the crystals, and show a similar tendency to self-associate in solution. Binding studies by bio-layer interferometry reveal cooperative binding of very short (VS)-core-plus-O-antigen polysaccharide (COPS) to the periplasmic domains of both proteins, but with decreased affinity for the A107P mutant. Our studies reveal that subtle and localized structural differences in PCPs can have dramatic effects on LPS chain-length distribution in bacteria, for example by altering the affinity for the substrate, which supports the role of the structure of the growing Oag polymer in this process.

## Introduction

*Shigella flexneri* (*S*. *flexneri*) is an important human pathogen that causes diarrhoea, fever, and shigellosis (bacillary dysentery), which is a potentially life-threatening bacterial infection of the colon. The heterogeneous serospecificity of *S*. *flexneri* is defined by the O-antigen (Oag), which is a component of lipopolysaccharide (LPS), a glycolipid distributed on the outer membrane surface of bacterial cells. LPS is comprised of three regions: (1) the Oag, consisting of a number of oligosaccharide repeats; (2) the core oligosaccharides; and (3) lipid A, which anchors the LPS to the membrane [1].

Three different Oag biosynthetic pathways have been characterized in different bacteria: the Wzy-dependent, the ATP binding cassette (ABC) transporter-dependent, and the synthase-dependent pathways [2]. *S*. *flexneri* uses the Wzy-dependent pathway [3], which is the pathway also used for the synthesis of other glycans such as the capsular polysaccharides [4]. The Oag repeat unit in *S*. *flexneri* is a tetra-saccharide, comprising three rhamnose sugars and one *N*-acetylglucosamine sugar. Biosynthesis of Oag initially occurs on the cytoplasmic side of the inner membrane and, as a result of a series of successive glycosyl transferase reactions, Oag repeat units are assembled onto the undecaprenyl phosphate lipid carrier, and then moved across the inner membrane by the Wzx flippase to the periplasmic side. The polymerization of Oag repeat units is subsequently carried on by the Wzy polymerase, forming an Oag polymer [5,6]. The Oag polymer chains are neither all of the same length nor of random length, and fall in a defined range termed the modal distribution. This distribution is regulated by two Wzz proteins in *S*. *flexneri*: WzzB_SF_, which confers an average modal chain-length of 10–17 Oag repeat units (short (S)-type), and Wzz_pHS2_, which confers a very long (VL-type) modal chain-length of > 90 Oag repeat units [7,8]. In the final step of LPS biosynthesis, the Oag polymer is transferred from the undecaprenyl phosphate to the oligosaccharide core of lipid A by the WaaL ligase and the entire LPS structure is then transported to the outer membrane (by the Lpt system) [9].

LPS acts as an important virulence factor against host defences, and the loss of its Oag modal chain-length regulation in *S*. *flexneri* has been shown to cause a defect in IcsA-mediated actin-based motility [10,11], and to affect complement resistance [12]. Distinctive LPS modal chain-lengths in *S*. *flexneri* also confer different degrees of resistance to colicin E2 [13], a bacterial plasmid-encoded DNase produced to kill competing bacteria [14].

The polysaccharide co-polymerases (PCPs) that control Oag length distribution fall into three classes [15]. The *S*. *flexneri* Wzz proteins are members of the PCP1a family and are located in the inner membrane. They contain a hydrophilic periplasmic polypeptide segment, and two trans-membrane (TM) regions at the N- and C-terminal ends. Despite low sequence similarities between PCP1a family proteins, the individual protomers have a comparable three-dimensional architecture, and self-assemble into a bell-shaped quaternary structure [16,17]. However, different oligomeric sizes have been reported for different PCP1a family proteins. Crystal structures of the periplasmic domains suggested that the PCP1a family proteins WzzB_ST_ (from *Salmonella enterica* serovar Typhimurium), and WzzE and Wzz^FepE^ (from *Escherichia coli*) form pentameric, octameric, and nonameric structures, respectively [16]. However, electron microscopy data was inferred to correspond to hexameric structures for full-length WzzB_ST_, Wzz^FepE^ and Wzz_K40_ [17]. The crystal structure of the periplasmic domain of WzzB_SF_ has recently been shown to form an open trimer, while chimeric hybrid protein variants based on WzzB_SF_ and WzzB_ST_ instead formed octameric barrels [18,19]. Finally, investigation of full-length *E*. *coli* WzzB and WzzE by blue-native gel analysis, size-exclusion chromatography coupled to multi-angle light scattering (SEC-MALS), electron microscopy and crystallography suggested octameric stoichiometry for both proteins [20]. It remains unclear if the oligomeric states of Wzz proteins relate to Oag length distributions.

Site-directed and in-frame insertion mutagenesis studies of WzzB_SF_ revealed that mutations in the TM or periplasmic regions can influence the Oag modal chain-lengths [21–23]. Some WzzB_SF_ mutants display shortened or lengthened Oag modal chain-length distribution, while other mutants display no change in Oag modal chain-length distribution or completely inactivate WzzB_SF_. Studies of chimeric WzzB_ST_ and WzzB_SF_ molecules support the concept that variations in several different protein regions can have an effect on Oag modal chain-length [21,23]. Taken together, these observations imply that differences in Oag length distributions imparted by Wzz proteins are dictated predominantly by the amino acids exposed at specific regions of the inner and outer surface of the oligomeric proteins.

To shed further light on the mechanism of chain-length regulation, we performed a structural and biochemical analysis of WzzB_SF_ and its site-directed mutant WzzB_SF_^A107P^, which was identified by a random mutagenesis approach. The residue 107 is located in a helix in the base-domain of the protein, close to the trans-membrane regions. WzzB_SF_^A107P^ confers a shortened Oag chain-length to bacteria and leads to a loss of resistance to colicin E2. Binding analyses reveal positive cooperativity for the interaction of very-short (VS) core-plus-Oag polysaccharide (COPS) with the periplasmic domain of WzzB_SF_, and a decreased COPS-binding affinity for the A107P mutant. The crystal structure of the mutant protein shows only subtle structural differences near the mutation, compared to the wild-type protein. Our observations suggest that a single amino-acid substitution on the inner surface of the periplasmic domain can lead to significant effects on the regulation on Oag modal chain-length, and implicates the LPS-binding affinity as an important parameter in the mechanism of this process.

## Materials and Methods

### Bacterial strains and growth conditions

The *S*. *flexneri* strain RMA4053 serotype Y *wzz*::*kan* ^*r*^, cured of the virulence plasmid and pHS-2, and carrying pCDFDuet-1 (Novagen), is from our laboratory collection [13,24]. The *Escherichia coli* strain XL10 Gold (*endA1 glnV44 recA1 thi-1 gyrA96 relA1 lac Hte* Δ*(mcrA)183* Δ*(mcrCB-hsdSMR-mrr)173 tet* ^*R*^ *F'[proAB lacI* ^*q*^ *Z*Δ*M15 Tn10(Tet* ^*R*^ *Amy Cm* ^*R*^ *)]*) was obtained from Stratagene. These strains were routinely grown at 37°C in Luria-Bertani (LB) broth (10 g/l tryptone, 5 g/l yeast extract, 5 g/l NaCl) with aeration for 16 to 18 h. Cultures were then diluted 1/20 into fresh broth, induced with 0.01 mM (w/v) isopropyl-β-D-thiogalactopyranoside (IPTG) and grown for 4 h at 37°C to an optical density 600 nm (OD_600_) of ~1. Antibiotics were used at the following concentrations: 100 μg/ml ampicillin, 50 μg/ml kanamycin and 100 μg/ml streptomycin.

### Construction of recombinant DNA plasmids

The construction of the plasmid encoding WzzB_SF_^A107P^ was performed as follows. Random mutagenesis with GeneMorph II EZClone Domain kit (Stratagene) was employed to produce a number of the mutant constructs (not shown) following the manufacturer’s instructions. The plasmid pRMCD30, a pQE30 (Qiagen)-based construct containing the *wzz*_*SF*_ opening reading frame [21], was incubated with Mutazyme II DNA Polymerase (Agilent) for 30 cycles to generate mutant megaprimers GL1 (5’-AGAGTAGAAAATAATAATGTTTCTGG-‘3) and GL2 (5’-CTTCGCGTTGTAATTACGC-‘3). The purified megaprimers were then incubated with pRMCD30 and EZClone enzyme for 25 cycles. The resultant PCR products were subjected to *Dpn*I treatment and introduced into *E*. *coli* XL10-Gold competent cells by transformation. Extracted plasmid DNA from pooled transformants was electroporated into *S*. *flexneri* Y RMA4053 *wzz*::*kan* ^*r*^ using a Bio-Rad GenePulser (according to the manufacturer’s instructions) and, following screening for an altered LPS profile by SDS-PAGE and silver staining, one isolate with a shortened LPS Oag modal chain-length was selected. The DNA was isolated, re-transformed into *E*. *coli* XL10-Gold and subjected to DNA sequencing with pQE30-specific primers: #Promoter Region (5’-CCCGAAAAGTGCCACCTG-3’) and #Reverse Sequencing (5’-GGTCATTACTGGAGTCTTG-3‘). The resultant plasmid pQE30::*wzzB*_*SF*_ [A107P] harbours a single alanine-to-proline amino-acid substitution at residue 107 (coding for the WzzB_SF_^A107P^ protein) and was chosen for further analysis in this study. For protein crystallization purposes, the *wzzB*_*SF*_ and its mutant *wzzB*_*SF*_ [A107P] were subcloned into an N-terminal hexahistidine tag-containing vector, pMCSG7 [25], by the ligation-independent cloning (LIC) method using the forward and reverse primers 5’-TACTTCCAATCCAATGCC-GAGAAATGGACGTCAACAGC-3’ and 5’–TTATCCACTTCCAATGTTATTTCGGACTATCGCGACG -3’, respectively. Plasmid DNA extraction was performed using the QIAprep Spin Miniprep kit (Qiagen). All constructs were verified by DNA sequencing at the Australian Genome Research Facility.

### Recombinant protein expression and purification

The periplasmic domain of WzzB_SF_ (residues 54–295) or the equivalent construct containing the A107P mutation (WzzB_SF_^A107P^), both containing N-terminal hexa-His tags, were expressed using the autoinduction method [26] in *E*. *coli* BL21(DE3) cells (Life Technologies). The cells were grown at 37°C for approximately 16 h after induction and then harvested by centrifugation. For protein purification, the harvested cells were resuspended in the pre-chilled lysis buffer (50 mM HEPES pH 8.0, 400 mM NaCl, 20 mM imidazole, 1 mM DTT, 1 mM PMSF, and 5% glycerol) and lysed using a digital sonifier (Branson). The cell debris and insoluble material were removed by centrifugation at 15,000 x *g* and 4°C. The resulting supernatant was collected and loaded onto a 5 ml HisTrap FF column (GE Healthcare), which was pre-equilibrated with the lysis buffer. To remove unbound proteins and contaminants, the column was washed with 20 column volumes of the lysis buffer. The protein was eluted with the buffer containing 50 mM HEPES pH 8.0, 400 mM NaCl, 250 mM imidazole, 1 mM DTT, 1mM PMSF, and 5% glycerol. The collected sample was concentrated to 2 ml using a 10 kDa molecular-weight cutoff Amicon (Millipore) and then diluted in cleavage buffer (50 mM HEPES pH 8.0, 200 mM NaCl, 1 mM DTT, 0.5 mM EDTA, 5% glycerol) to a volume of 20 ml. To remove the N-terminal His-tag, tobacco-etch-virus (TEV) 3C protease (0.5 mg/ml) was added to the sample in 1:100 protease:protein molar ratio for overnight digestion. The digested protein sample was loaded back onto the HisTrap FF column to capture the protein sample without the His-tag. The protein was purified further by size-exclusion chromatography (Superdex 200 HiLoad 26/60, GE Healthcare) in the size-exclusion buffer consisting of 10 mM HEPES pH 8.0, 200 mM NaCl, 5% glycerol and 1 mM DTT. Fractions from the peak were analysed by SDS-PAGE and the purest fractions were pooled together and concentrated in the size-exclusion buffer using Amicon. The concentrated protein was flash-frozen in liquid nitrogen before storing at -80°C.

### Protein crystallization

The initial crystallization conditions of WzzB_SF_ and its A107P mutant were obtained using commercial screens from Hampton Research and Molecular Dimensions. Crystals from these initial conditions gave poor diffraction and therefore further optimization was carried out by screening with a citric-acid/HEPES/CHES (in 2:3:4 ratio) buffer system from pH 4 to 10 [27], and a wide range of additives. The X-ray diffraction data were collected on the WzzB_SF_ and WzzB_SF_^A107P^ protein crystals, both grown in 0.1 M citric-acid/HEPES/CHES buffer (pH 7.3), 18% PEG 400, 18% PEG 8000 and 0.1 M MgCl_2_. The concentration of protein used for crystallization was 12–13 mg/ml according to Bradford assays. All crystallization trials were performed by hanging drop vapor diffusion at 18°C using 1 μl of protein and 1 μl of reservoir solution in the drop, suspended over 0.5 ml reservoir solution.

### X-ray diffraction data collection and crystal structure determination

X-ray diffraction data were collected using MX1 and MX2 beamlines at the Australian Synchrotron. The crystals were cryo-protected by transferring to the reservoir solution supplemented with 20% (v/v) glycerol, and flash-cooled by immersion in liquid nitrogen. Data were integrated and scaled using XDS [28] and XSCALE [29]. The data from the crystals of the wild-type and mutant proteins were used to a resolution of 2.50 and 2.47 Å, respectively, based on CC_1/2_ analysis (Table 1). Both structures were determined using the structure of WzzB_ST_ (PDB code: 3B8P [16]) as a search model for molecular replacement with the program PHASER [30]. Model building was completed manually with COOT [31] and refined with BUSTER [32]. The structures were validated using Molprobity [33]. Data collection and refinement statistics are summarized in Table 1.

**Table 1:** Crystallographic data.

Protein crystal (PDB code)	WzzB_SF_ (4ZM1)	WzzB_SF_^A107P^ (4ZM5)
Diffraction source	MX1, Australian Synchrotron	MX1,usMX2, Australian Synchrotron
Wavelength (Å)	0.9537	0.9537
Temperature (K)	100	100111 100
Detector	ADSC Quantum 210r	ADSC Quantum 315r
Crystal-to-detector distance (mm)	265	350
Rotation range per image (°)	0.5	1
Total rotation range (°)	180	245
Exposure time per image (s)	3	1
Space group	P12_1_1	P12_1_1d P12_1_1
Unit-cell parameters (Å, °)	*a* = 80.89, *b* = 61.31, *c* = 90.86 α = 90.00, β = 94.21, γ = 90.00	*a* = 80.57, *b* = 62.46, *c* = 90.24 α = 90.00, β = 94.06, γ = 90.00
Resolution range (Å)	31.68–2.55 (2.641–2.55)	32.14–2.48 (2.569–2.48)
Total No. of reflections	122216	179710
No. of unique reflections	28733 (2780)	31224 (3119)
Completeness (%)	98.24 (96.36)	97.7 (98.6)
Multiplicity	3.8 (3.8)	3.2 (3.2)
<*I*/σ(*I*)>	9.4 (1.4)	7.2 (1.5)
*R*_meas_ ^1^	0.132 (1.319)	0.186 (1.149)
*R*_p.i.m_ _._ ^2^	0.068 (0.673)	0.099 (0.614)
<I> half-set correlation CC(1/2)	0.997 (0.612)	0.99 (0.598)
Overall *B* factor from Wilson plot (Å^2^)	52.88	48.63
R_work_/^4^R_free_ (%) ^3^	19.33/22.45	19.51/24.20
No. of atoms (protein/water)	5633/58	5556/141
R.m.s. deviations		
Bond length (Å)/bond angle (°)	0.01/1.7	0.01/1.7
Ramachandran plot (%)^4^		
Favored	97.78	98.69
Allowed	2.02	1.16
Outliers	0.2	0.15

Values in parentheses are for the highest resolution shell.

^1^ *R*_meas_ =.Σ_*hkl*_{1/[1(*hkl*)-1]}^1/2^Σ_*i*_ | I_*i*_(*hkl*)-(I(*hkl*))|/Σ_*hkl*_Σ_i_ I_i_(*hkl*).

^2^ *R*_p.i.m._ = Σ_*hkl*_{1/[N(*hkl*)-1]}^1/2^Σ_*i*_ | I_*i*_(*hkl*)-(I(*hkl*))|/Σ_*hkl*_Σ_i_ I_i_(*hkl*).

^3^ *R*_work_ = Σ_*hkl*_(||Fobs_*hkl*_|-|Fcalc_*hkl*_||)/|Fobs_*hkl*_|, where |Fobs_*hkl*_| and |Fcalc_*hkl*_| represent the observed and calculated structure factor amplitudes. *R*_free_ is equivalent to *R*_work_ but calculated using 5% of the reflections not used in refinement.

^4^ Calculated using Molprobity [33].

### Multi-angle light scattering (MALS)

MALS experiments were carried out using a DAWN HELEOS II 18-angle light scattering detector coupled with an Optilab rEX refractive-index detector (Wyatt Technology), combined inline with a Superdex 200(10/300) GL (GE Healthcare) size-exclusion column connected to a Prominence UFLC chromatography system (Shimadzu). One hundred μl of the proteins in a serial dilution (20, 10, 5, and 2.5 mg/ml) were applied to the size-exclusion column with a flow rate of 0.5 ml/min. The buffer for all experiments corresponded to 20 mM Tris-HCl pH 7.5 and 200 mM NaCl.

### Small-angle X-ray scattering (SAXS)

Purified WzzB_SF_ and WzzB_SF_^A107P^ were thawed and dialysed for 18 h into a buffer containing 10 mM HEPES (pH 7.50), 150 mM NaCl and 1 mM DTT, at 4°C. Data was collected at the SAXS/WAXS beamline of the Australian Synchrotron, on a Pilatus 1M detector at a sample-to-detector distance of 1.5 m and a wavelength of 1.12713 Å, yielding a range of momentum transfer 0.011 < *q* < 0.500 Å^-1^, where *q* = 4π.sin(*θ*)/*λ*. Five successive 1:2 dilutions were prepared for each protein using reserved dialysis buffer. For each sample, 90 μL was injected through a 1.5 mm diameter quartz capillary at 298 K, at a rate of 1 μL/s with image capture occurring every 1 s. Consistent, successive exposures were normalized to transmitted intensity, reduced, scaled to absolute intensity using pure water, then averaged and buffer-subtracted. Pre- and post-sample buffer exposures were compared to check for radiation-induced aggregate build-up. Evidence of poor buffer subtraction was observed in the highest two concentrations of WzzB_SF_^A107P^, and these were manually scaled with respect to the buffer scattering at 0.3 Å^-1^ < *q* < 0.5 Å^-1^. All datasets were subsequently restricted to points where *q* < 0.2 Å^-1^. Data reduction and subtraction were performed using the beamline’s in-house software, Scatterbrain. (http://www.synchrotron.org.au/index.php/aussyncbeamlines/saxswaxs/software-saxswaxs). The ATSAS 2.6.0 software package was used for further analysis (51, 52). *R*_g_ and *I*(0) were calculated from Guinier analysis of the data region where *q*.*R*_g_ <1.3, using AUTORG in PRIMUS (53). *P*(*r*) distributions were obtained for all constructs by indirect transformation in GNOM (54), informed by AUTOGNOM.

### LPS SDS-PAGE and silver staining

LPS was prepared as previously described [22,34]. Cells (1 x 10^9^), grown and induced as described above, were harvested by centrifugation. The cell pellet was resuspended in the buffer containing 10% (w/v) glycerol, 2% (w/v) SDS, 4% (w/v) β-mercaptoethanol (β-ME), 0.1% (w/v) bromophenol blue, 1 M Tris-HCl, pH 7.6, and incubated with 2 μg/ml proteinase K for 16 h. The isolated LPS samples were electrophoresed on 15% SDS-polyacrylamide gels for 13 to 14 h at 12 mA. The gels were stained with silver nitrate and developed with formaldehyde [34].

### COPS purification and determination of concentration

LPS was purified from RMA2159 (*S*. *flexneri* 2457T cured of the virulence plasmid) and RMA4328 (*S*. *flexneri* 2457T *wzz*::*kan* ^*R*^ */pHS2*::*tn5-cml* ^*R*^ strain cured of the virulence plasmid and carrying pRMCD76 [35]) as described in [36]. Purified LPS was then hydrolysed by heating in 1% (v/v) acetic acid for 90 min at 100°C, followed by ultracentrifugation at 142,000 x *g* for 5 h at 4°C. The supernatant containing the COPS was freeze-dried and stored at 4°C. The COPS molarity concentration was determined using the bicinchoninate assay [37] and maltose was used to construct a standard curve.

### Protein SDS-PAGE and western immunoblotting

Bacteria were grown and induced as described above, harvested by centrifugation, and resuspended in 1x sample buffer [38]. Protein samples were heated at 100°C for 5 min, except for the cross-linking samples, which were heated at 60°C for 5 min, before loading onto 12% or 15% SDS-polyacrylamide gels. The electrophoresed protein samples were transferred to a nitrocellulose sheet (Medos), which was further subjected to incubation with polyclonal anti-WzzB_SF_ antibodies [21] at 1/500 dilution. Detection of the proteins was performed with goat anti-rabbit horseradish-peroxidase-conjugated antibodies (KPL) and chemiluminescence reagent (Sigma). The BenchMark protein ladder (Invitrogen) was used as molecular-mass standards.

### Chemical cross-linking analysis

Cells grown and induced as described above were harvested (5 x 10^8^ cells) by centrifugation, resuspended in the chilled cross-linking buffer (10 mM potassium phosphate/10 mM Tris at pH 6.8) and incubated with (and without for controls) 0.5% (v/v) formaldehyde (Univar) in the cross-linking buffer at 25°C for 1 h. Both cross-linked and control samples were washed once with buffer, resuspended in 1x sample buffer, and heated to 60°C for 5 min prior loading onto 12% SDS-polyacrylamide gels. The gels were subjected to western immunoblotting with anti-WzzB_SF_ antibodies as described above.

### Colicin spot sensitivity assay

Colicin was prepared and the colicin spot sensitivity assays were performed as previously described [13].

### Protein-LPS binding affinity measurements

Bio-layer interferometry using the BLItz^TM^ instrument (Pall Life Sciences) was used for binding affinity experiments. WzzB_SF_ or WzzB_SF_^A107P^, containing an N-terminal His-tag (see the “Recombinant protein expression and purification” section) was immobilized onto a nickel-charged NTA biosensor and subjected to 10 min of rehydration in the reaction buffer (20 mM Tris-HCl, pH 7.5, 500 mM NaCl, 0.008% Triton X-100, and 0.09% Tween-20) before binding experiments. High concentrations of NaCl and Tween-20 (non-ionic surfactant) were used to reduce non-specific binding of VS-COPS to the biosensor. The immobilization of His-tagged proteins to the sensor was performed with 4 μl of 2 μM Wzz protein in the drop holder for 120 s, and followed by 30 s incubation of the sensor in the reaction buffer. For the association step, the biosensor was incubated with 4 μl of different concentrations of VS-COPS for a period of 5 s. If the association was allowed for 60 s, the binding kinetics displayed a bi-phasic behaviour, possibly due to consecutive binding events or modification of the substrate (S1A Fig). Within 5 s, the first association event reached a transient equilibrium, allowing the calculation of R_eq_ (biosensor signal when the binding between Wzz variants and VS-COPS is at equilibrium) for the first and main binding phase (S1B Fig). The dissociation step consisted of the incubation of the biosensor with 250 μl of the reaction buffer for 60 s. Representative raw data for Wzz variants as a function of VS-COPS concentration are presented as S2 Fig. Each concentration of VS-COPS was analyzed in triplicate. For the calculation of the R_eq_ value, the biosensor binding signal value (Y) was plotted as a function of time (X), and the R_eq_ value was derived from the signal difference between the control and the experiment, when the transient equilibrium was reached (~5 s). The K_d_ value was obtained by fitting the data to the allosteric sigmoidal equation: Y = X^h*R_max_ /(X^h+K_d_); X: concentration of VS-COPS in molar units; Y: R_eq_; h: Hill coefficient (>1, resulting in a sigmoidal curve due to positive cooperativity) [39].

### Ethics statement

The anti-WzzB_SF_ antibody was produced under the National Health and Medical Research Council (NHMRC) Australian Code of Practice for the Care and Use of Animals for Scientific Purposes and was approved by the University of Adelaide Animal Ethics Committee.

## Results

### WzzB_SF_^A107P^ confers a shortened LPS Oag modal chain-length

**Fig 1:**
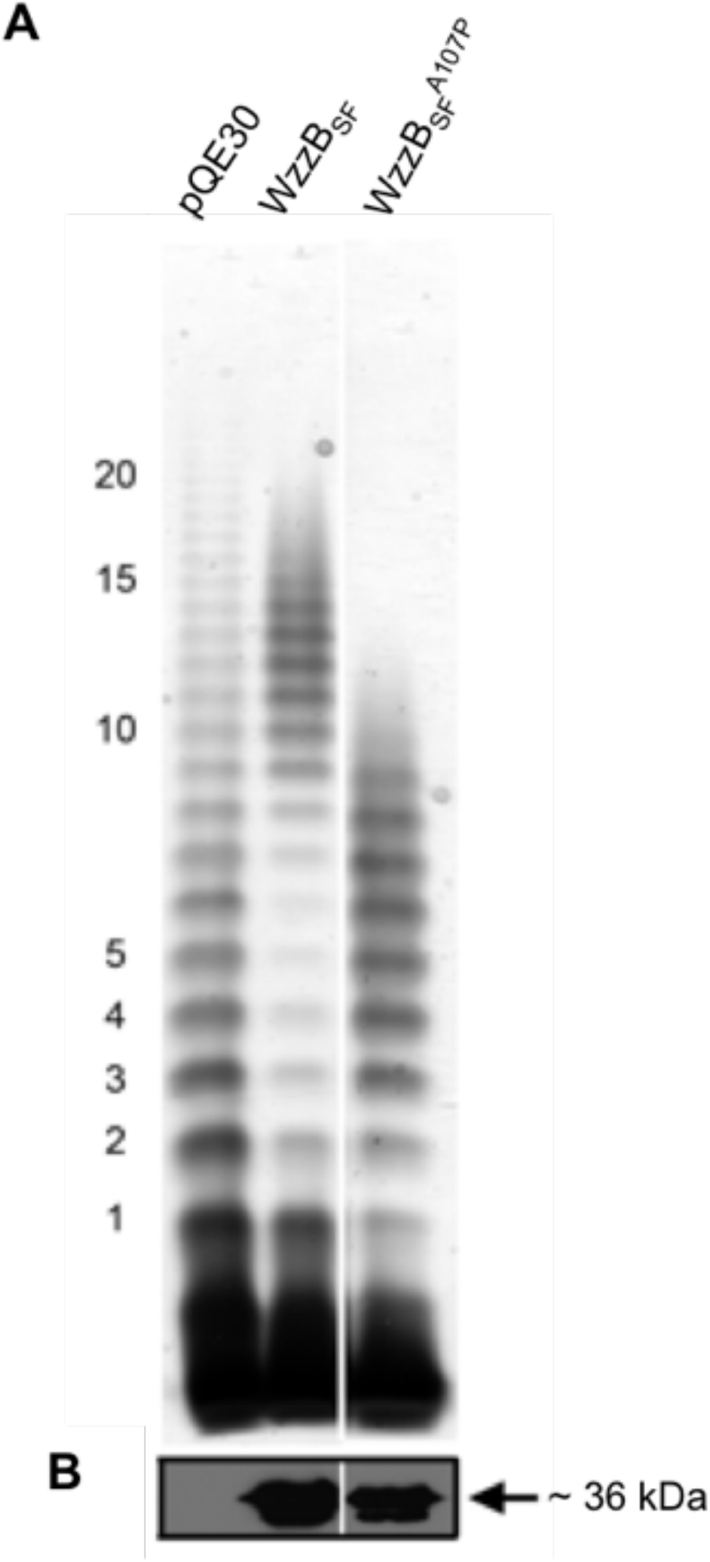
LPS analysis of *S*. *flexneri* expressing wild-type WzzB_SF_ or the WzzBSFA107P mutant. Whole-cell lysates of *S*. *flexneri* RMA4053 strains expressing either wild-type WzzB_SF_ or the WzzB_SF_^A107P^ mutant proteins were (A) treated by proteinase-K and electrophoresed on a SDS 15% polyacrylamide gel, followed by detection of LPS by silver-staining (the first 20 Oag repeat units are indicated); or (B) electrophoresed on SDS 15% polyacrylamide gels and then subjected to western immunoblotting with WzzB_SF_ polyclonal antibodies. The size of the full-length WzzB_SF_ protein (~36 kDa) is indicated. Each lane corresponds to 5 x 10^7^ bacterial cells.

Plasmids pRMCD30 (coding for wild-type WzzB_SF_), pQE30::*wzzB*_*SF*_[A107P] (coding for WzzB_SF_^A107P^) and the empty vector pQE30 were transformed into *S*. *flexneri* RMA4053 and LPS extracted from the transformed strains was analyzed by silver staining. The results showed the expected S-type LPS Oag modal chain-length of 10–17 repeat units conferred by wild-type WzzB_SF_, but a shortened LPS Oag modal chain-length of 2–10 Oag repeat units conferred by WzzB_SF_^A107P^ (Fig 1A). The control strain pQE30 showed the expected broader Oag chain-length distribution (Fig 1A). Western immunoblotting on whole-cell lysates with anti-WzzB_SF_ antibodies detected proteins of ~36 kDa for both WzzB_SF_ and WzzB_SF_^A107P^ (Fig 1B).

### WzzB_SF_^A107P^ confers reduced resistance to colicin E2

**Fig 2:**
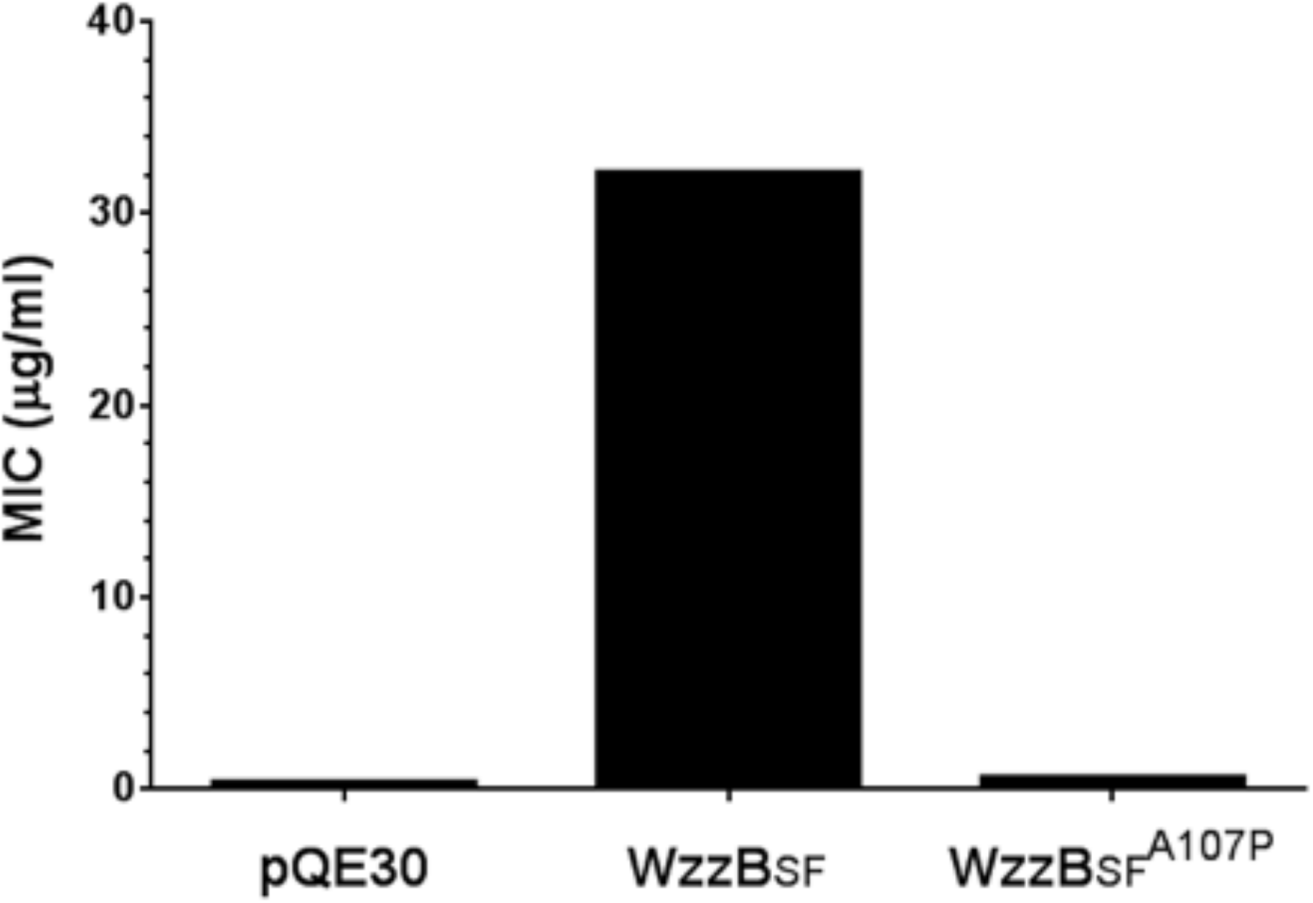
Analysis of colicin E2 sensitivity of WzzBSFA107P. Spot sensitivity assays with purified colicin E2 were performed using *S*. *flexneri* RMA4053 strains expressing either WzzB_SF_ or WzzB_SF_^A107P^ [13]. The minimum inhibitory concentration (MIC) of colicin E2 (in μg/ml) required to generate a clear zone of bacterial growth inhibition is shown on the *y-*axis (*n* = 3). There was no difference in MIC values between replicates for each particular strain. The MIC for each strain expressing pQE30, WzzB_SF_ and WzzB_SF_^A107P^ were 0.25, 32 and 0.5 μg/ml, respectively.

The Oag modal chain-length has previously been shown to affect sensitivity by *S*. *flexneri* to colicin E2 [13]. To investigate the colicin E2 resistance of *S*. *flexneri* expressing WzzB_SF_^A107P^, spot sensitivity assays were performed. Strains expressing pQE30 and WzzB_SF_^A107P^ exhibited high sensitivity to colicin E2 (minimum inhibitory concentration [MIC] ≤ 0.5 μg/ml), while the strain expressing wild-type WzzB_SF_ showed resistance to colicin E2 (MIC = 32 μg/ml) (Fig 2). These results are consistent with previous data showing that LPS Oag modal chain-lengths of less than 10–17 repeat units (such as that conferred by WzzB_SF_^A107P^) correlate with reduced resistance to colicin E2 [13].

### VS-COPS binds cooperatively to the periplasmic domains of WzzB_SF_ and WzzB_SF_^A107P^

**Fig 3:**
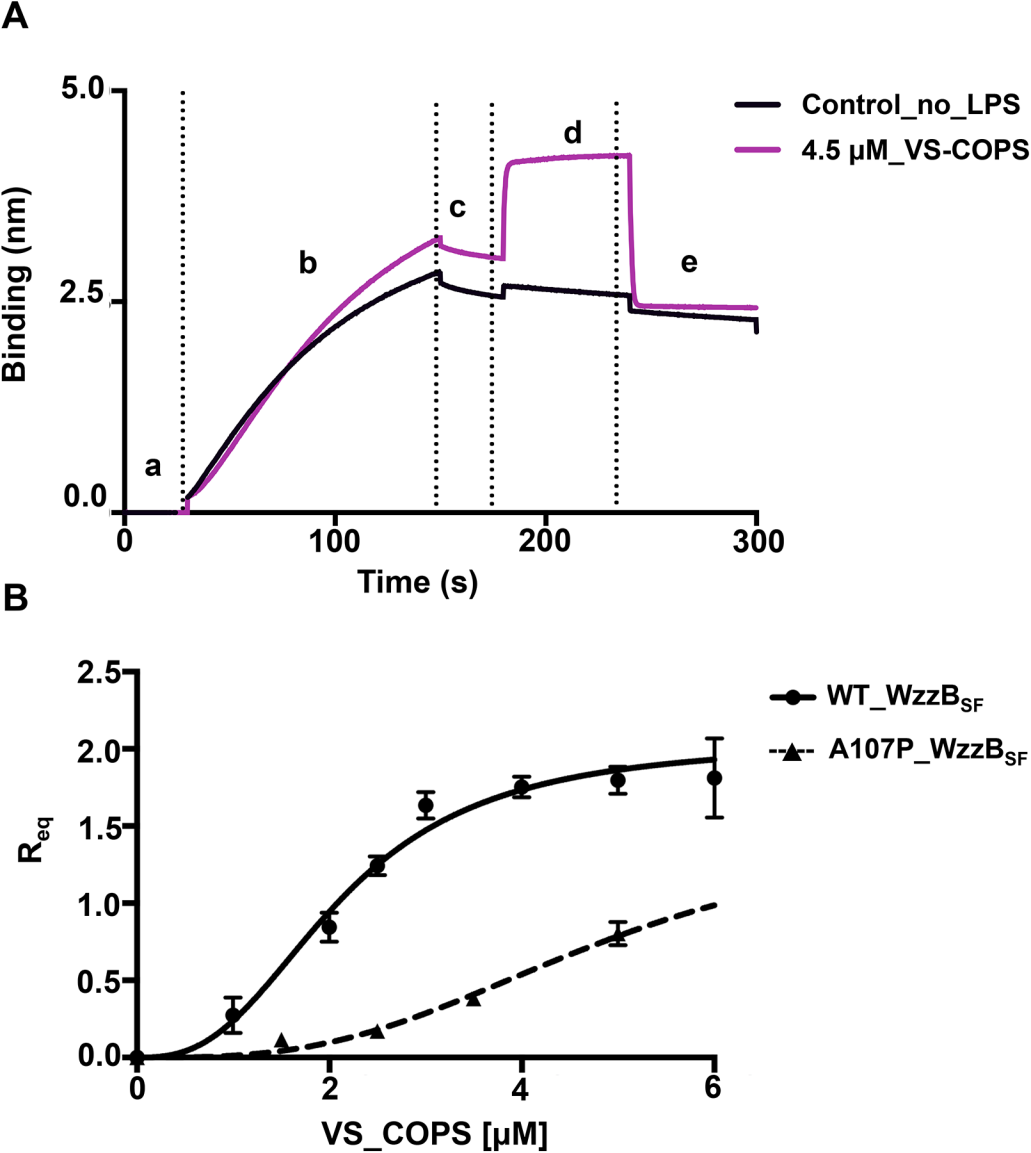
VS-COPS binding measurements. (A) Raw data for the binding between the His-tagged periplasmic domain of WzzB_SF_ and VS-COPS. The experiment comprised 5 steps: (a) initial baseline stabilization (30 s); (b) loading of 2 μM of His-tagged WzzB_SF_ to the Ni-NTA biosensor (120 s); (c) stabilization of the baseline with the reaction buffer (30 s); (d) loading of 4.5 μM VS-COPS (association, purple curve) or the reaction buffer alone (control, black curve) (60 s); and (e) wash with reaction buffer (disassociation, 60 s). The increase of signal during the loading of His-tagged WzzB_SF_ to the Ni-NTA sensor (Step b) indicates that the binding was effective. 4.5 μM VS-COPS gave a significant binding signal at the association step (step d), compared to the control. (B) R_eq_ values, indicating the biosensor signal shift induced by the binding of WzzB_SF_ (solid line) and WzzB_SF_^A107P^ (dashed line) to VS-COPS at equilibrium (see the Materials and Methods section), were fitted with the Hill equation for cooperative binding (Y = X^h*R_max_ /(X^h+K_d_)), yielding a Hill coefficient of 2.6, which demonstrates positive cooperativity. The binding of His-tagged WzzB_SF_^A107P^ and VS-COPS could not be analysed at high VS-COPS concentrations due to non-specific binding of VS-COPS to the sensor (see the Materials and Methods section).

The effect that the A107P mutation may have on the polymerization of Oag tetra-saccharide repeat units was investigated by determining the binding affinities between Wzz variants and VS-COPS. The experiment assumed that due to the short length, VS-COPS can adequately replace tetra-saccharides in a binding experiment. Direct label-free affinity assay using bio-layer interferometry was conducted to measure the differences in binding affinities between the periplasmic domains of WzzB_SF_ and WzzB_SF_^A107P^, and VS-COPS (Fig 3A). The association curves showed that the binding process is bi-phasic (S1 Fig), which could be caused by the heterogeneity of the VS-COPS sample and the nature of COPS binding to Wzz. As the first binding event generates the most important signal, the analysis was restricted to the first 5 seconds of the association (S1 and S2 Figs). The data fitted well to the sigmoidal Hill equation for allosteric binding, with the Hill coefficient greater than 1 implying that positive cooperativity occurs in the binding between Wzz proteins and VS-COPS. The *K*_d_ values corresponded to 2.12 ± 0.11 μM and 4.9 ± 0.17 μM for the wild-type and the A107P mutant proteins, respectively, showing that the mutation has a negative effect on the binding. An average Hill coefficient of ~3.0 was obtained for both the wild-type and mutant proteins.

### Crystal structures of the periplasmic domains of WzzB_SF_ and the WzzB_SF_^A107P^ mutant

**Fig 4:**
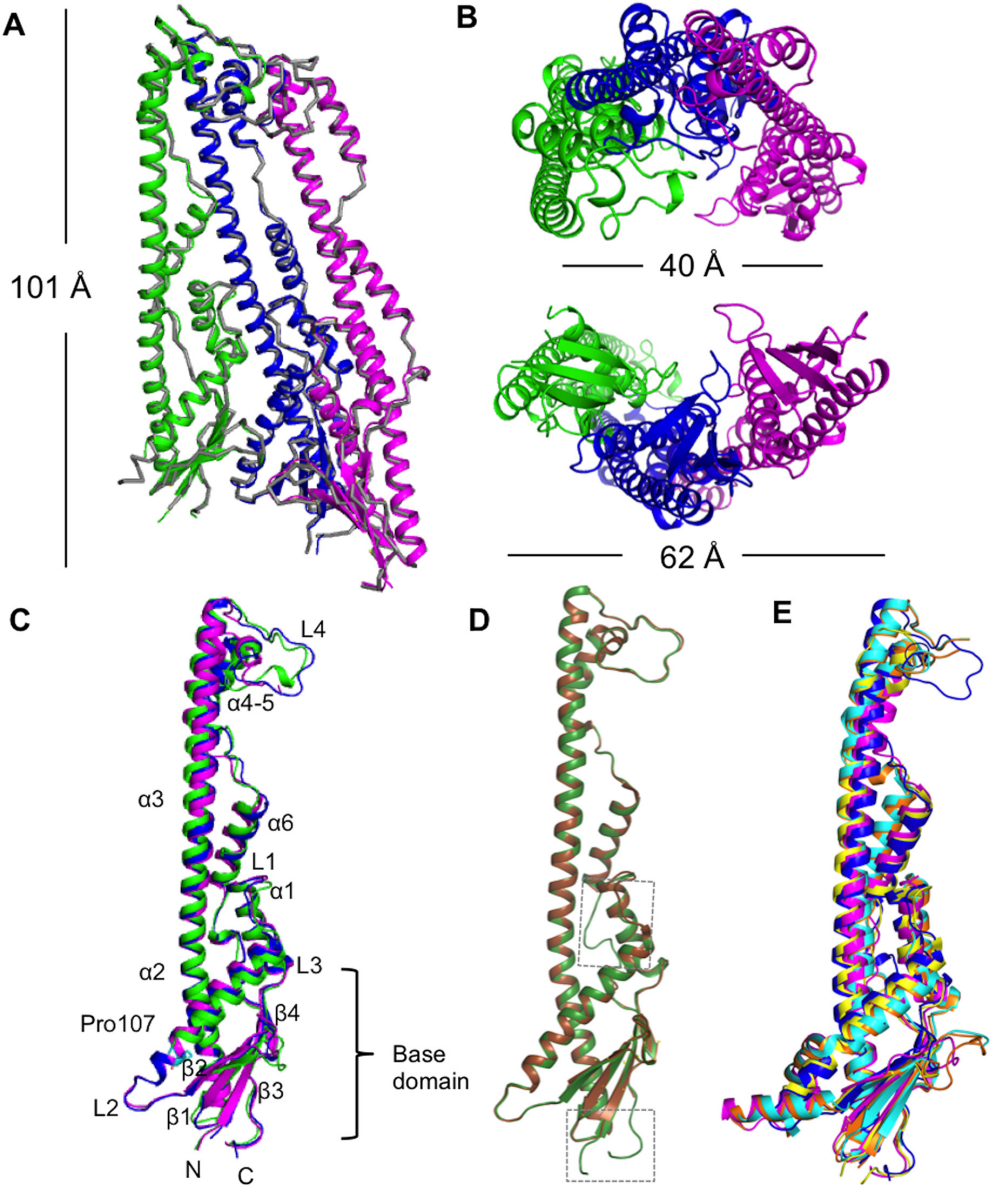
Crystal structures of the periplasmic domains of WzzB_SF_ and WzzBSFA107P. (A) Superimposition of the trimeric structure of the WzzB_SF_^A107P^ periplasmic domain (subunits A, B and C are shown in green, blue and magenta cartoon representation, respectively) onto the wild-type WzzB_SF_ structure (shown in grey ribbon representation), yielding an r.m.s.d. of 0.52 Å for 644 Cα atoms. The height of the trimer is approximately 102 Å. (B) Top- and base-view of the WzzB_SF_^A107P^ trimeric structure. The width across the top/base region is around 40/62 Å. (C) Superimposition of three protomers of the periplasmic domain of WzzB_SF_^A107P^. The residue proline-107 is shown in cyan stick representation. The elements of secondary structure are labelled. (D) Superimposition of subunit A from WzzB_SF_ determined in this work (shown in green cartoon representation; subunit B) with WzzB_SF_ determined previously (PDB ID 4E2H, in brown; subunit B) [18]. The key differences between the structures are found in the α2 helix, the loop connecting the α5 helix and β4 strand and the regions at the termini; these regions are highlighted with dashed-line rectangles. Our WzzB_SF_ structure allows the visualization of all residues between amino acid 53/55 and 289/290 in different chains of the trimer; on the other hand, several loop and terminal regions could not be modelled in different chains in the 4E2H structure, including the residues 271–273 in any of the three chains. (E) Superimposition of the central B subunit from WzzB_SF_^A107P^ (shown in blue cartoon representation) with Wzz_ST_ (PDB ID 3B8P, in yellow); Wzz^FepE^ (3B8N, in pink); FepE_O157 (3B8M, in orange); WzzE (3B8O, in magenta) [16,18].

The crystals of both wild-type and mutant WzzB_SF_ proteins were obtained under identical conditions. Both periplasmic domains form an open trimer architecture of approximately 102 Å in height, and 40 Å and 62 Å in width across the top and the base, respectively (Fig 4). The structures of the trimers of WzzB_SF_ and WzzB_SF_^A107P^ are highly similar with a root-mean-square distance (r.m.s.d.) of 0.52 Å for 640 Cα atoms. The residue 107 is located in the α2 helix in the α/β base domain, where it would be in close proximity to the trans-membrane regions of the protein (Fig 4). The largest structural differences between the wild-type and mutant proteins are found in the flexible loop regions, especially the L4 region at the top of the structure. There is only a subtle local disruption of the α2 helix because of the A107P mutation.

### Self-association of WzzB_SF_ proteins

**Fig 5:**
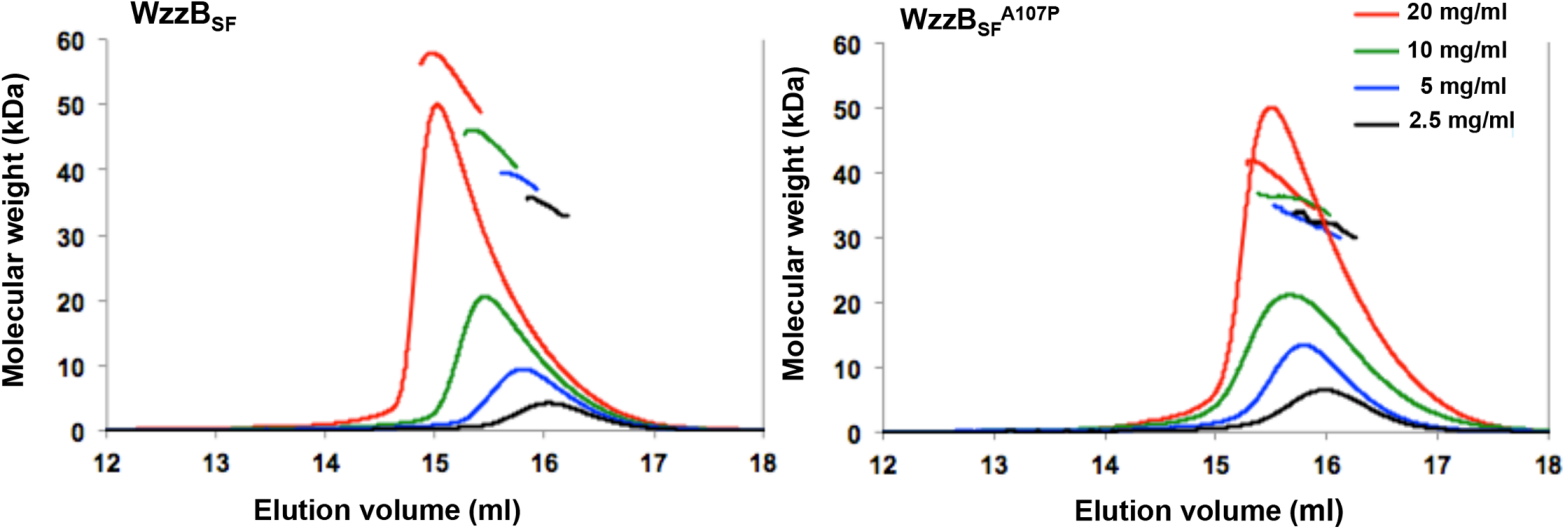
Multi-angle laser light scattering analysis of the periplasmic domains of WzzB_SF_ and WzzBSFA107P. The periplasmic domains of the wild-type WzzB_SF_ (left panel) and WzzB_SF_^A107P^ proteins (right panel) were subjected to SEC-MALS analyses with concentrations (mg/ml) of 20 (red line), 10 (green line), 5 (blue line), and 2.5 (black line). The chromatograms indicate the trace from the refractive index detector during SEC (x-axis: elution volume (ml)). The lines above or under the peaks correspond to the averaged molecular weight (Mw; y axis) distribution across the peak determined by MALS. Theoretical molecular weight of a monomer is 27.5 kDa.

The oligomeric states of the periplasmic domains of WzzB_SF_ and the A107P mutant in solution were investigated by SEC-MALS at protein concentrations between 20 and 2.5 mg/ml. This analysis (Fig 5) indicates a dynamic equilibrium between monomers and oligomers in a concentration-dependent manner, for both the wild-type and mutant proteins. The A107P mutant showed a slightly lower degree of self-association than the wild-type counterpart at increased protein concentrations.

**Fig 6:**
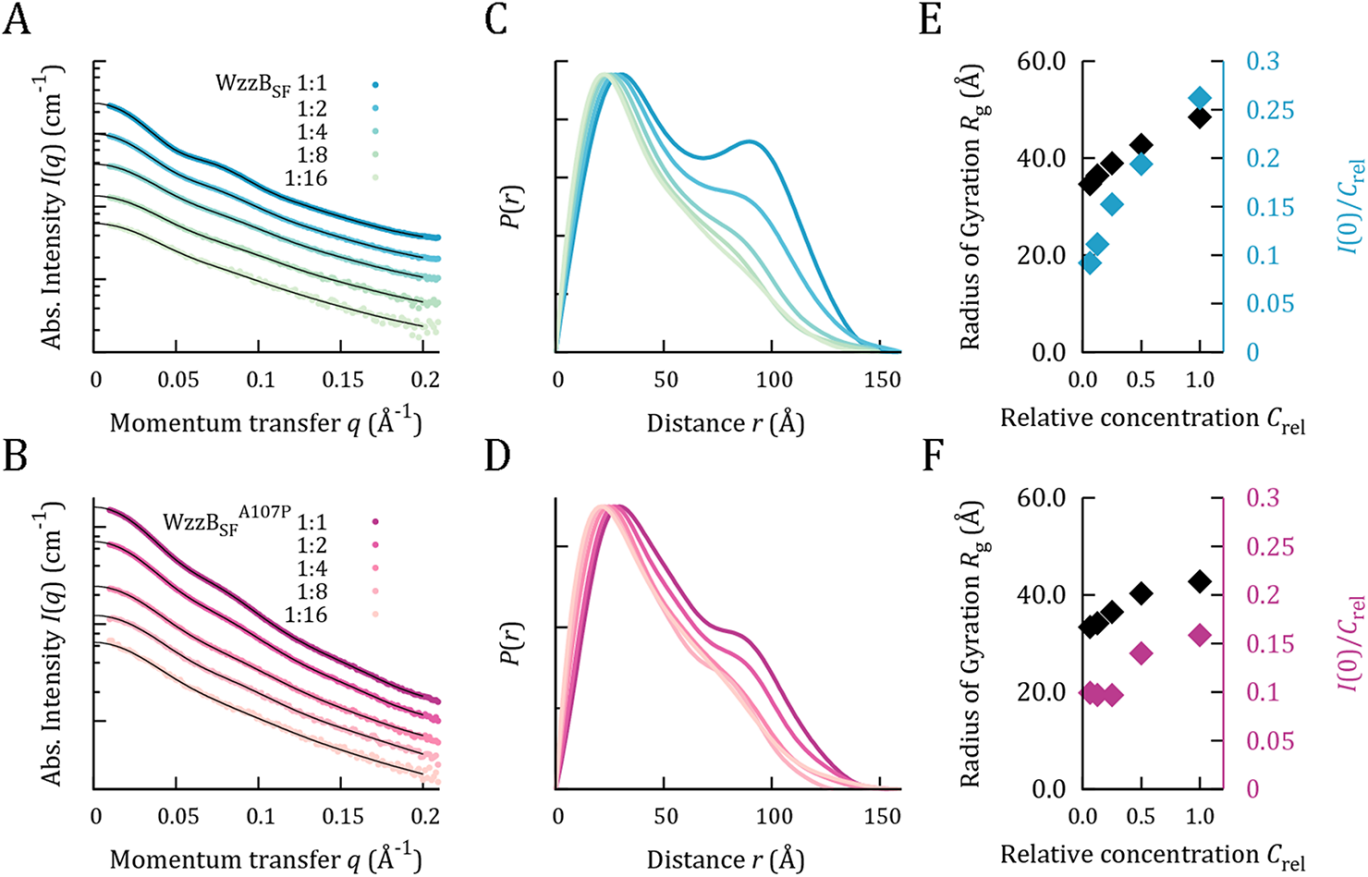
Small-angle X-ray scattering (SAXS) data. (A-B) Averaged and subtracted SAXS data for a dilution series of WzzB_SF_ (A) and WzzB_SF_^A107P^ (B), placed on absolute scale by comparison to the scattering of pure water. Data points are shown as circles coloured by dilution. The fits of the corresponding *P*(*r*) distributions are shown as black lines. (C-D) Distance distributions calculated from the scattering data by indirect Fourier transformation, showing the frequency, *P*(*r*), of intermolecular distances of length *r* within each particle. Distributions are calculated from each dilution in the concentration series. Plots have been scaled to the maximum of the highest concentration for ease of visualisation. Top panel, WzzB_SF_; bottom panel, WzzB_SF_^A107P^. Considerable concentration dependence can be observed in both, particularly in the highest concentration of the wild-type protein. (E-F) Analysis of concentration-dependence of particle size and total scattering. Radius of gyration, *R*_*g*_ (black diamonds), and the ratio of zero-angle scattering to concentration, *I*(0)/C_rel_ (coloured diamonds) are plotted for each dilution of each protein. Both metrics are calculated from Guinier analysis of the low-*q* region of the data, and would be expected to be stable for a monodisperse, non-interacting species. Approximate non-relative concentrations are 10 mg/ml for 1:1 WzzB_SF_, and 5 mg/ml for 1:1 WzzB_SF_^A107P^.

Self-association in the periplasmic domains of WzzB_SF_ and WzzB_SF_^A107P^ was also examined by small-angle X-ray scattering (SAXS). Clear concentration dependence was observed for both WzzB_SF_ and WzzB_SF_^A107P^ (Fig 6). This was apparent in the emergence of a second maximum in the distance distributions at high concentration, and an increase in *I*(0)/*c* with increasing concentration. Guinier plots are linear, suggesting that this is not due to non-specific aggregation (S3 Fig). Thus, the data indicates that the protein is subject to concentration-dependent self-association into oligomers. Unfortunately, precise sample concentrations could not be determined prior to this experiment, and the higher concentrations of WzzB_SF_^A107P^ were also affected by buffer mismatch. As a result, we do not present molecular weights or shape analysis. Concentration-dependence is nonetheless obvious, particularly for WzzB_SF_. Shifts in 1:1 and 1:2 WzzB_SF_^A107P^ may be exacerbated by the buffer mismatch, although change is also evident as emerging features from 0.05 < *q* < 0.1 Å^-1^.

**Fig 7:**
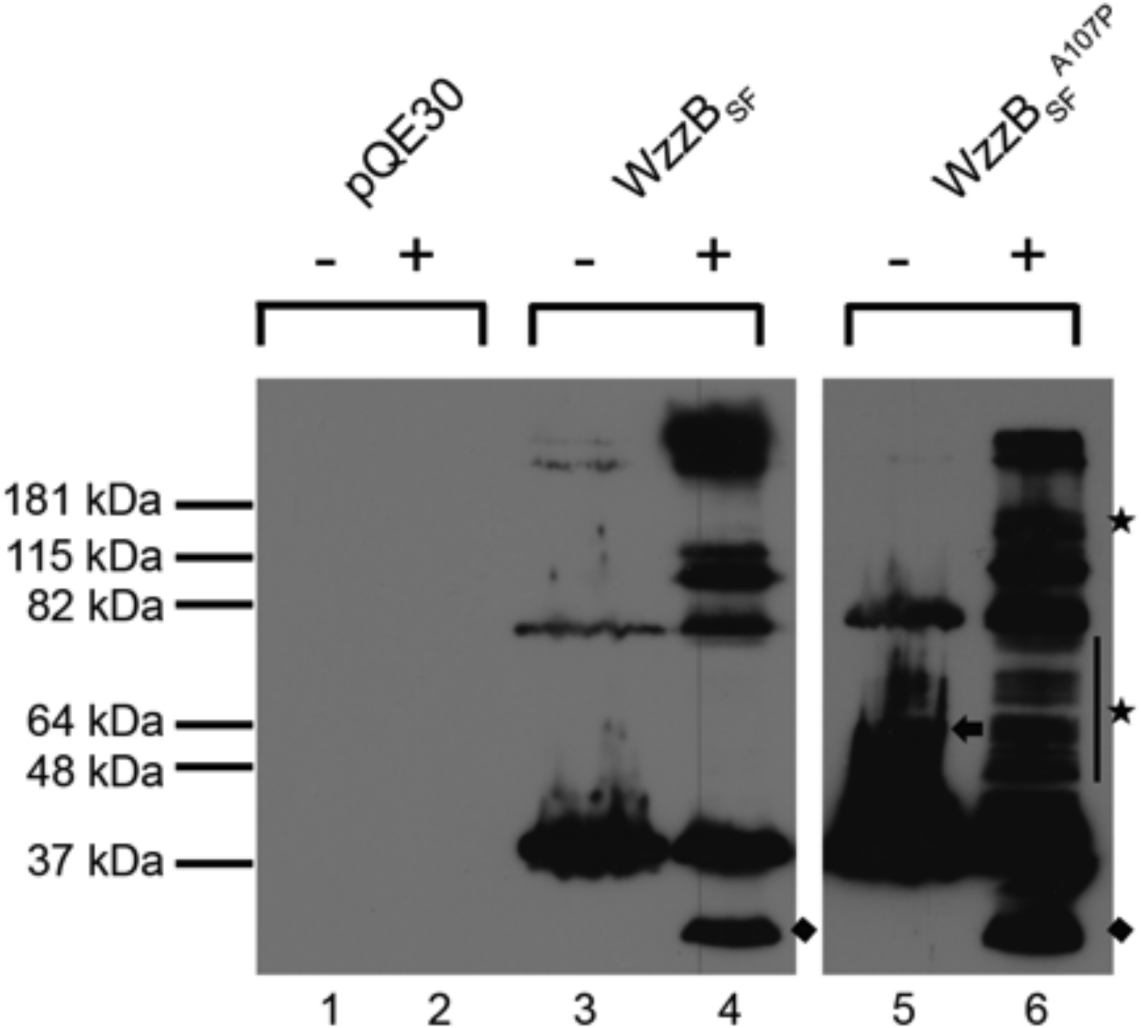
Formaldehyde cross-linking analysis. *S*. *flexneri* RMA4053 strains expressing either WzzB_SF_ or WzzB_SF_^A107P^ were harvested, resuspended in potassium phosphate buffer, and treated with 0.5% formaldehyde (+) at 25°C; controls were incubated without formaldehyde (-). Cells were resuspended in the sample buffer and heated at 60°C for 5 min, electrophoresed on a SDS 12% polyacrylamide gel, followed by western immunoblotting with WzzB_SF_ antibodies. The black arrow indicates the extra band present in WzzB_SF_^A107P^ non-cross-linked sample; the stars indicate the extra bands present in WzzB_SF_^A107P^ cross-linked sample; and the diamonds indicate the ~30 kDa form of WzzB_SF_ that has been reported previously [22]. Each lane corresponds to ~5 x 10^7^ bacterial cells.

To test the ability of full-length WzzB_SF_ and WzzB_SF_^A107P^ to form higher-order oligomers, cross-linking was performed on whole-cell samples with 0.5% formaldehyde (Fig 7). Wild-type WzzB_SF_ showed readily detectable monomeric (36 kDa), dimeric (72 kDa) and what appeared to be a faint higher molecular mass band (>180 kDa) in non-cross-linked samples (Fig 7, lane 3). The cross-linked sample revealed bands at ~30 kDa, ~36 kDa, ~82 kDa, a doublet at ~115 kDa and a strong higher molecular-mass doublet band at >180 kDa, which indicated the presence of higher-order oligomerization (Fig 7, lane 4). The non-cross-linked WzzB_SF_^A107P^ sample showed similar results to the wild-type WzzB_SF_, with the exception of an additional band at ~ 64 kDa (Fig 7, lane 5, indicated by a black arrow). The cross-linked WzzB_SF_^A107P^ revealed all bands present in the cross-linked WzzB_SF_, with an additional band at ~150 kDa and several bands between 37 and 82 kDa in size (Fig 7, lane 6, indicated by black stars), illustrating WzzB_SF_^A107P^ had a cross-linking profile different from the wild-type WzzB_SF_. The low molecular mass ~30 kDa band present in both the WzzB_SF_ and WzzB_SF_^A107P^ cross-linked samples (Fig 7, lanes 2 & 4, indicated by black diamonds) has been reported previously to correspond to a different conformation of WzzB_SF_ [22]. In summary, the formaldehyde cross-linking suggests that the mutant protein can form a higher proportion of differently sized oligomers compared to the wild-type counterpart.

## Discussion

To investigate the critical determinants of the regulation of LPS Oag chain-length by PCP1a family proteins in Gram-negative bacteria, we carried out functional and structural studies on *Shigella flexneri* WzzB_SF_. The Wzz proteins regulate the polymerization of LPS Oag before ligation to the lipid A core in the process of LPS biosynthesis [40]. Several models have been proposed previously for the Wzz function. In the molecular-clock model, elongation was suggested to occur for a defined period of time before switching to ligation [41]. In the chaperone model, Wzz was proposed to act as a molecular chaperone controlling the ratio of polymerase Wzy interacting with the ligase WaaL [15]. In the organizing-scaffold model, the oligomeric state of Wzz was suggested to control the number of associated Wzy molecules [16]. In the molecular ruler model, the Wzz periplasmic barrel was proposed to measure the Oag length [16,42]. A recent study of the WbdA and WbdD proteins, acting in ABC transporter-dependent assembly of LPS in *Escherichia coli* O9a, revealed that the polymer size is indeed controlled by the length of a coiled-coil domain that acts as a molecular ruler [43]. Finally, in the chain-feedback model, the growing Oag polymers were proposed to adopt higher-order structures affecting Wzz binding, conferring a limited range of lengths [19]. Unfortunately, none of these models is consistent with all the available data. Islam and Lam have therefore recently proposed a hybrid model, combining the chain-feedback and molecular-ruler mechanisms [3].

In some of the models described above, the size of the oligomer of the PCP1a family proteins plays a crucial role. The oligomeric structures have been analysed in recent studies using a plethora of techniques including x-ray crystallography, electron microscopy and small-angle X-ray scattering (SAXS) [16–18,20,44–46]. Different oligomeric structures have been observed among the family members, and through the use of different techniques. The reasons could include the presence or absence of trans-membrane regions, and variations in experimental conditions that may not mimic the natural environment of the bacterial inner membrane. Reconstitutions of the full-length proteins in a lipid bilayer, mimicking the bacterial membrane, have in fact been reported to show consistent quaternary structures for various Wzz homologues [17,20], although these studies reached different conclusions regarding the stoichiometry. These discrepancies complicate our understanding of the native oligomeric states of Wzz proteins, and give rise to uncertainty regarding the relationship between the oligomeric sizes and Oag chain-length regulation.

Mutational studies of PCP1a family members have shed further light on the corresponding mechanisms of Oag chain-length regulation. The wild-type *E*. *coli* Wzz^FepE^ protein and its loop-4 mutant confer distinct modal chain-length distributions, despite highly similar structures of the protomers and oligomers [18,23]. This observation suggests that specific regions of Wzz proteins could mediate the Oag chain-length distribution, rather than their oligomeric states. Indeed, site-directed mutagenesis revealed that single amino-acid changes of the Wzz proteins from *E*. *coli* and *S*. *flexneri* can cause variations of the Oag chain-length [21,47]. Likewise, the LPS chain-length distribution of two strains of *E*. *coli* differs significantly, although the corresponding Wzz proteins share 90% sequence identity [48]. A random in-frame linker mutagenesis study of WzzB_SF_ yielding proteins with 5-amino-acid insertions revealed five classes of mutants with different effects on Oag chain-length distributions [22]. The “class-II” mutants conferred shorter LPS Oag chains of 2–10 repeat units and showed fewer high-molecular-mass oligomers through formaldehyde cross-linking. By contrast, mutation of residue T321 of the Wzz2 protein from *Pseudomonas aeruginosa* resulted in a shortened Oag modal length, but an increased proportion of higher-order oligomers of the protein in the cross-linking analysis [42].

In the current study, we identified a mutant of *Shigella flexneri* WzzB_SF_, using a random mutagenesis approach, that causes a shortened Oag chain-length distribution. To investigate how this A107P mutation affects the regulation of the Oag chain-length distribution, we characterized the mutant protein biochemically and structurally. The wild-type and mutant proteins both display concentration-dependent self-association in solution, although the A107P mutation affects the self-association ability based on SEC-MALS and cross-linking. The crystal structures of the periplasmic domains of the wild-type and mutant proteins reveal an open trimer structure. In the WzzB_SF_^A107P^ structure, the mutation of Ala107 to proline causes a subtle local disruption to the α2 helix, which is located in the base domain. Proline is known for its distinctive rigidity compared to other amino acids, and its helix-breaking or kinking character. Although Ala107 is not located in the oligomeric interface of WzzB_SF_, the disruption of the α2 helix leads to some loss of contacts between the α2 helix and the neighboring β-strand and may affect the stability of the base domain and the oligomer. The mutation maps to the inside of the open trimer, consistent with most other residues affecting chain-length control that appear to be located on the inner surface of the periplasmic domain [12,24].

The A107P mutant conferred a shortened Oag modal chain-length (2–10 repeat units) and showed reduced resistance to colicin E2, comparable to the class-II mutants identified through WzzB_SF_ insertion mutagenesis previously [22]. The class-II mutations predominantly appear to localize to the “upper” inner surface of the oligomer (α6-α8 helices), whereas the A107P mutation is located at the C-terminus of α2 helix in the base domain close to the trans-membrane regions. Distinct regions of the protein can therefore affect the Oag modal chain-length distribution. Chemical cross-linking of the A107P mutant showed a different cross-linking profile to the wild-type protein, including an addition of a high-molecular-mass band (~ 150 kDa). By contrast, the class-II insertion mutants showed no detectable high-molecular-mass oligomers [22]. The WzzB_SF_^A107P^ mutant appears more comparable with the *P*. *aeruginosa* Wzz2 protein conferring a shortened Oag modal length, which showed an increased proportion of higher-order oligomers in the cross-linking analysis [42]. Different mutations may therefore confer shortened chain-length distributions through different mechanisms.

Our SEC-MALS and SAXS analyses revealed a concentration-dependent self-association of both WzzB_SF_ and WzzB_SF_^A107P^ periplasmic domains. This is comparable to what was observed for the periplasmic domains of Wzz^FepE^, WzzE, and WzzB_ST_ by analytical ultracentrifugation and dynamic light scattering [16]. A slightly lower tendency to self-associate was suggested for WzzB_SF_^A107P^ by the SEC-MALS analysis. By contrast, formaldehyde cross-linking of the full-length WzzB_SF_ and WzzB_SF_^A107P^ proteins suggested the mutant protein could form larger oligomers than the wild-type counterpart. Our results indicate that studies of truncated proteins may not adequately reflect the oligomeric state of the full-length proteins.

While our work was in progress, Kalynych and coworkers reported the structure of the periplasmic domain of WzzB_SF_ (residues 54–293) at 2.8 Å resolution (PDB ID 4E2H) [18]. Our structure allows the visualization of several loop regions and regions at the termini that could not be modelled in the 4E2H structure [18]. The two WzzB_SF_ crystal structures were obtained using similar crystallization conditions and feature analogous packing of molecules in the crystals. They both show an analogous open trimeric association of WzzB_SF_ protomers in the crystals (r.m.s.d. values of 0.34, 0.39, 0.44 Å for 210, 198 and 182 Cα atoms for the central and the two edge subunits, respectively; r.m.s.d of 0.48 Å for 581 Cα atoms of the trimer). As already discussed by Kalynych and coworkers, the trimeric structure shows substantial differences from other available structures of Wzz proteins [16,18]. Superposition of the central subunit from our WzzB_SF_ trimer structure with the respective protomers from WzzB_ST_ (PDB ID 3B8P), Wzz^FepE^ (PDB ID 3B8N), WzzE (PDB ID 3N8O) and FepE_O157 (PDB ID 3B8M) yields r.m.s.d. values of 0.72 Å, 3.1 Å, 1.7 Å, and 2.7 Å for 136, 166, 137, and 145 Cα atoms, respectively (Fig 4D). The structural differences are significantly larger when superimposing the trimers of WzzB_SF_ with those of WzzB_ST_, Wzz^FepE^, WzzE and FepE_O157 oligomers [16,18] with r.m.s.d. values of 4.3 Å, 19.6 Å, 3.9 Å, and 5.4 Å for 432, 629, 459, and 489 Cα atoms, respectively, highlighting the different packing of subunits in the oligomers.

To date, the binding of LPS to Wzz proteins has not been characterized quantitatively, confined to co-immunoprecipitation, circular dichroism and SAXS studies [40,44,48]. We performed direct label-free quantitative affinity assays to assess the binding affinity between WzzB_SF_ and COPS (the polysaccharide component of LPS but lacking lipid A). Our data show positive cooperativity in the binding of VS-COPS, suggesting that multiple binding sites occur on WzzB_SF_. The cooperativity could be a result of conformational changes of the Wzz protein upon binding, in accordance with substrate-induced conformational changes of Wzz proteins suggested previously, based on SAXS studies [44]. Cooperative binding has also been observed in the binding of polycationic antibiotic polymyxin *B* to purified LPS from *P*. *aeruginosa* [49]. Importantly, the A107P mutation resulted in a 2–3 fold reduction of the binding affinity to COPS, suggesting a relation between binding affinities between Oag repeat units and Wzz proteins and the regulation of Oag chain-length distribution. This needs to be tested on other WzzB_SF_ mutants or chimeras that conferred altered LPS length distributions [18,21–23]. Future work will involve the investigation of these mutants.

In summary, the knowledge of the regulatory mechanism of LPS Oag chain-length modal distributions is essential for the understanding of the pathogenicity of bacteria and the host immune responses to infection. The complexity of the regulation of chain-length distributions suggests that diverse factors may be involved, including the oligomeric state and stability of the Wzz proteins, the interaction with the Wzy polymerase, and the molecular characteristics of the polysaccharide itself and its interaction with the protein machinery [3,16,42,50,51]. Our study demonstrates that a single point mutation (A107P) of WzzB_SF_ can lead to a distinct LPS chain-length distribution through a direct effect on LPS binding and only subtle effects on the structure of the Wzz protein. Through the demonstration of cooperative binding between LPS and Wzz, our study lends support to the involvement of the growing polysaccharide chain on the regulation of its length, and is consistent with the hybrid model where Wzz protein cooperates as a molecular ruler with the growing polysaccharide to achieve a modal length distribution.

### Accession numbers

The crystallographic coordinates and structure factors have been deposited in the Protein Data Bank (PDB) with ID 4ZM1 (WzzB_SF_) and 4ZM5 (WzzB_SF_^A107P^).

## Supporting Information

S1 Fig VS-COPS binding to WzzB_SF_. (A) The association (60 s) of VS-COPS to WzzB_SF_ indicates a bi-phasic binding mode. The first binding phase reaches saturation at approximately 5 s. The second binding phase shows slower association kinetics and does not reach equilibrium within the 60 s of the association. The dashed line shows the saturation plateau for first binding event. (B) Calculation of R_eq_ for the first binding phase (5 s) of VS-COPS to His-tagged WzzB_SF_.
(PDF)

S2 Fig Representative raw data for the binding between VS-COPS and WzzB_SF_ proteins. (A) The association (5 s) of His-tagged WzzB_SF_ to different concentrations of VS-COPS at the first binding phase, and (B) the corresponding disassociation (5 s). (C-D) The association (5 s) and the dissociation (5 s) steps of His-tagged WzzB_SF_^A107P^ at different concentrations of VS-COPS.
(PDF)

S3 Fig Guinier analysis of SAXS data. Guinier analysis of SAXS data for WzzB_SF_, top panel; and WzzB_SF_^A107P^, bottom panel. The first 25 points of each dataset are shown transformed as *q* ^2^ vs ln *I*(*q*). Linear regressions used for determination of *R*_g_ and *I*(0) are shown as black lines. Linearity in the fitted region is apparent for all datasets. These regions were determined using AUTORG. Larger ranges of points are selected for lower concentrations as selection is constrained to points where *q*.*R*_g_ < 1.3, and *R*_g_ increases with concentration for these samples.
(PDF)
