# Supplementary material for: Structural and Biochemical Analysis of a Single Amino-Acid Mutant of WzzBSF That Alters Lipopolysaccharide O-Antigen Chain Length in Shigella flexneri
Source: PLoS One. 2015 Sep 17;10(9):e0138266. doi: 10.1371/journal.pone.0138266 (PMC4574919; doi:10.1371/journal.pone.0138266)
Supplement: S1 Fig — (A) The association (60 s) of VS-COPS to WzzBSF indicates a bi-phasic binding mode. The first binding phase reaches saturation at approximately 5 s. The second binding phase shows slower association kinetics and does not reach equilibrium within the 60 s of the association. The dashed line shows the saturation plateau for first binding event. (B) Calculation of Req for the first binding phase (5 s) of VS-COPS to His-tagged WzzBSF. (PDF) [file pone.0138266.s001.pdf]

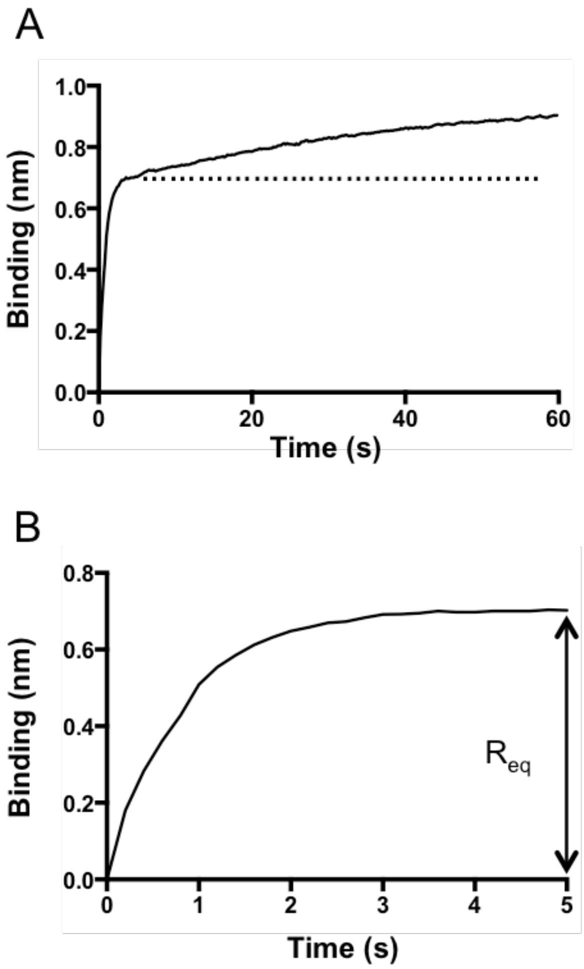

S1 Fig. VS-COPS binding to WzzB$_{SF}$. (A) The association (60 s) of VS-COPS to WzzB$_{SF}$ indicates a bi-phasic binding mode. The first binding phase reaches saturation at approximately 5 s. The second binding phase shows slower association kinetics and does not reach equilibrium within the 60 s of the association. The dashed line shows the saturation plateau for first binding event. (B) Calculation of R_{eq} for the first binding phase (5 s) of VS-COPS to His-tagged WzzB$_{SF}$.
