# Supplementary material for: Structural and Biochemical Analysis of a Single Amino-Acid Mutant of WzzBSF That Alters Lipopolysaccharide O-Antigen Chain Length in Shigella flexneri
Source: PLoS One. 2015 Sep 17;10(9):e0138266. doi: 10.1371/journal.pone.0138266 (PMC4574919; doi:10.1371/journal.pone.0138266)
Supplement: S2 Fig — (A) The association (5 s) of His-tagged WzzBSF to different concentrations of VS-COPS at the first binding phase, and (B) the corresponding disassociation (5 s). (C-D) The association (5 s) and the dissociation (5 s) steps of His-tagged WzzBSF A107P at different concentrations of VS-COPS. (PDF) [file pone.0138266.s002.pdf]

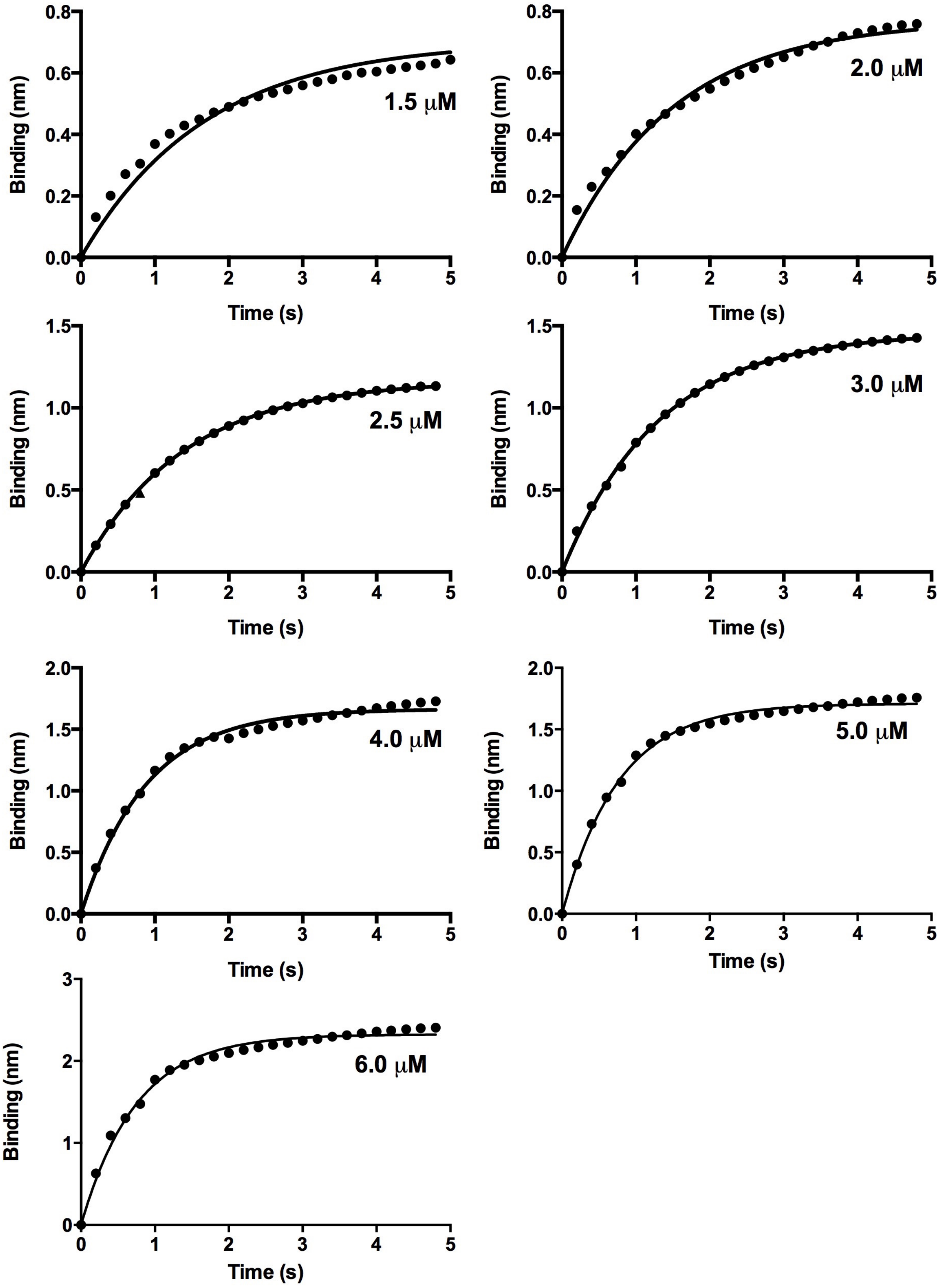

1.5 μM
2.0 μM
2.5 μM
3.0 μM
4.0 μM
5.0 μM
6.0 μM
Binding (nm)
Time (s)

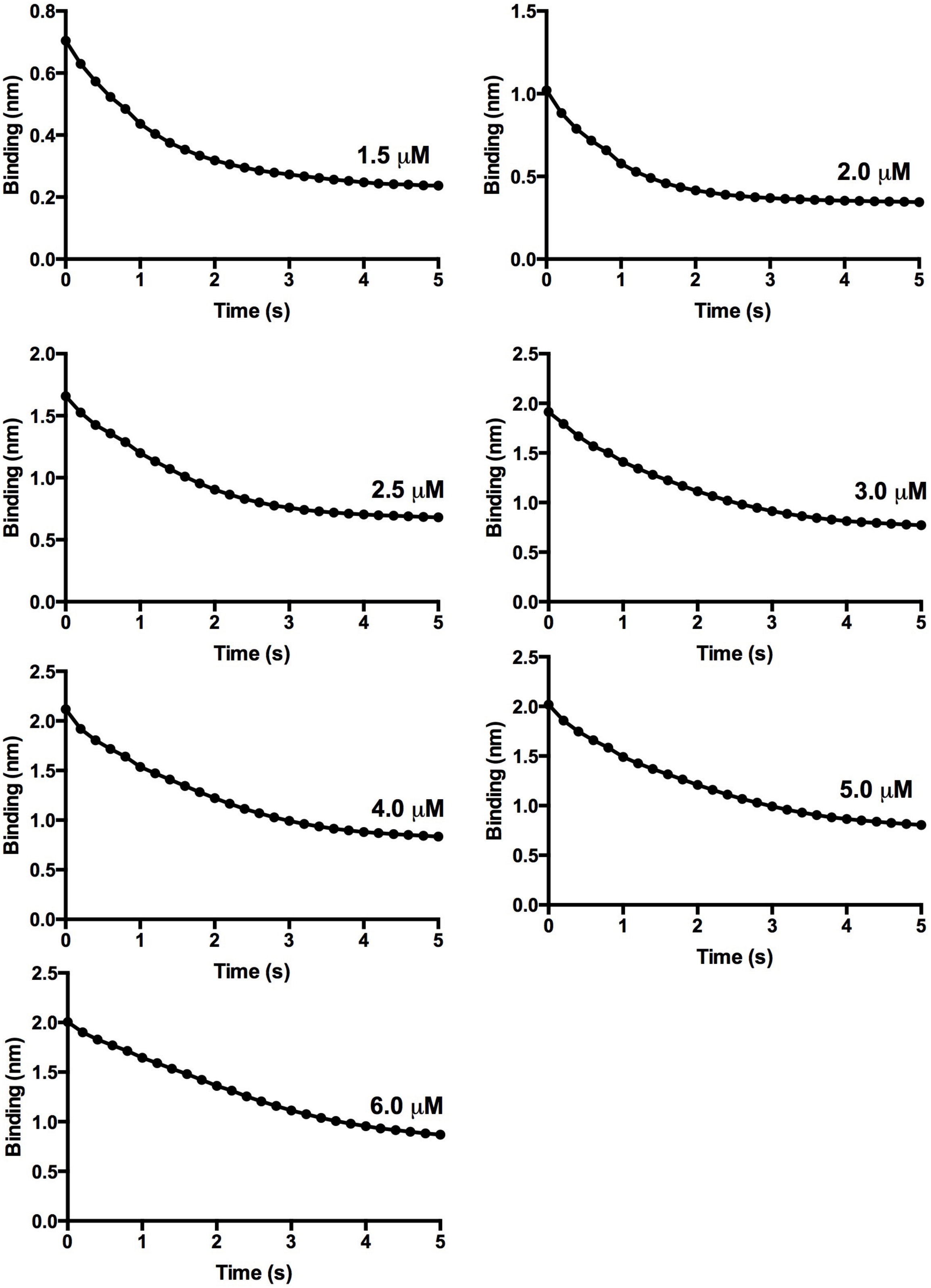

Binding (nm)
Time (s)
1.5 μM
2.0 μM
2.5 μM
3.0 μM
4.0 μM
5.0 μM
6.0 μM
0.0
0.2
0.4
0.6
0.8
0.5
1.0
1.5
2.0
2.5
0
1
2
3
4
5

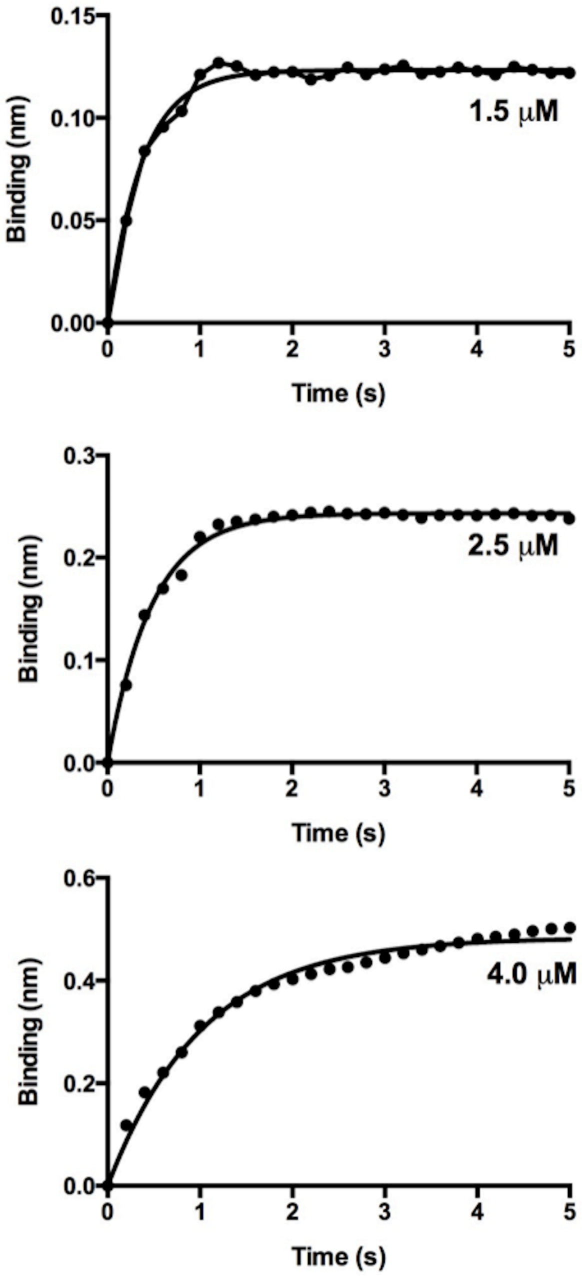
1.5 μM
2.0 μM
2.5 μM
3.5 μM
4.0 μM
5.0 μM
Binding (nm)
Time (s)

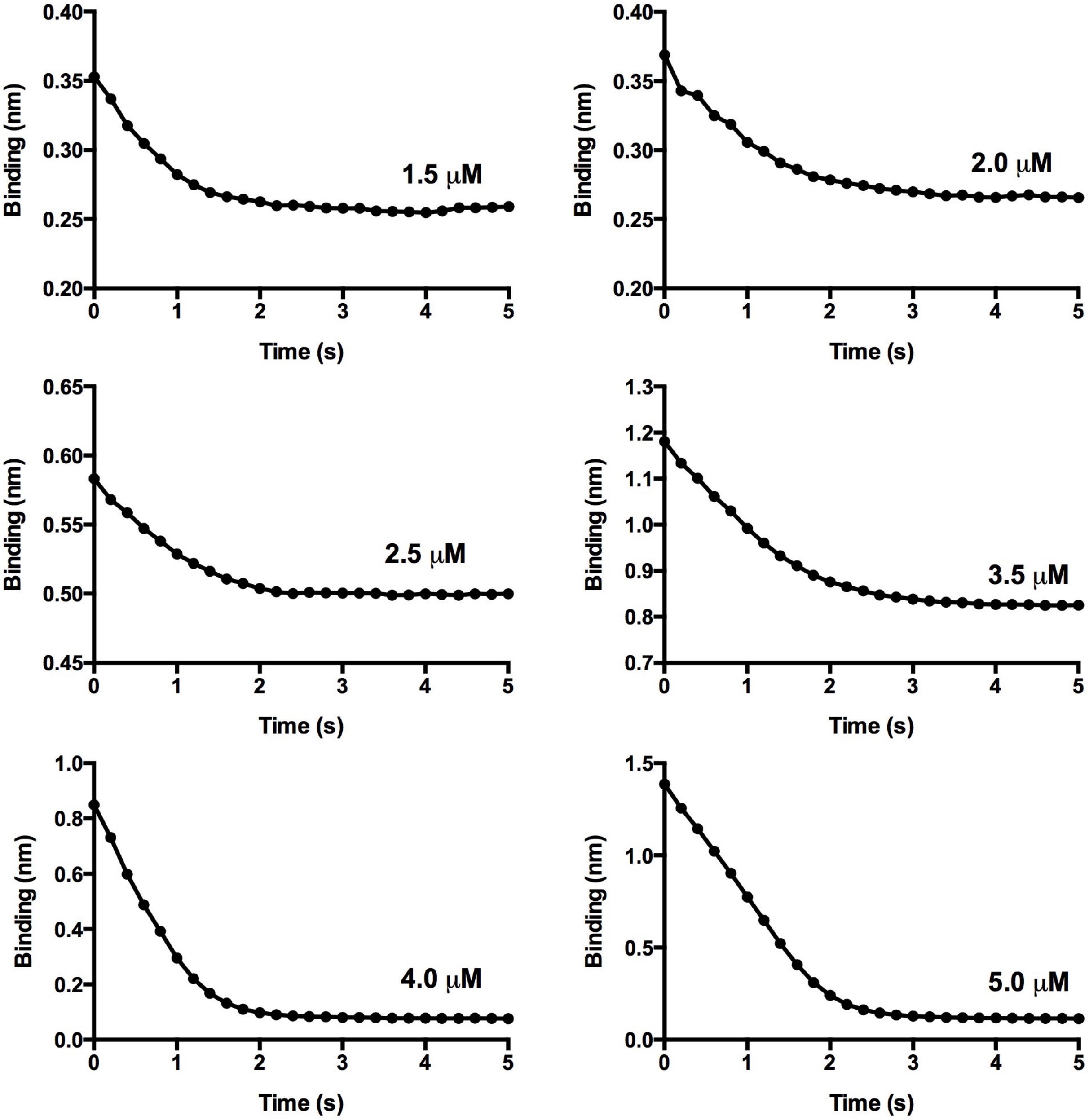
1.5 μM
2.0 μM
2.5 μM
3.5 μM
4.0 μM
5.0 μM
Binding (nm)
Time (s)

S2 Fig.. Representative raw data for the binding between VS-COPS and WzzB$_{SF}$ proteins. (A) The association (5 s) of His-tagged WzzB$_{SF}$ to different concentrations of VS-COPS at the first binding phase, and (B) the corresponding disassociation (5 s). (C-D) The association (5 s) and the dissociation (5 s) steps of His-tagged WzzB$_{SF}^{A107P}$ at different concentrations of VS-COPS.
