# Supplementary material for: Structural and Biochemical Analysis of a Single Amino-Acid Mutant of WzzBSF That Alters Lipopolysaccharide O-Antigen Chain Length in Shigella flexneri
Source: PLoS One. 2015 Sep 17;10(9):e0138266. doi: 10.1371/journal.pone.0138266 (PMC4574919; doi:10.1371/journal.pone.0138266)
Supplement: S3 Fig — Guinier analysis of SAXS data for WzzBSF, top panel; and WzzBSF A107P, bottom panel. The first 25 points of each dataset are shown transformed as q 2 vs ln I(q). Linear regressions used for determination of R g and I(0) are shown as black lines. Linearity in the fitted region is apparent for all datasets. These regions were determined using AUTORG. Larger ranges of points are selected for lower concentrations as selection is constrained to points where q.R g < 1.3, and R g increases with concentration for these samples. (PDF) [file pone.0138266.s003.pdf]

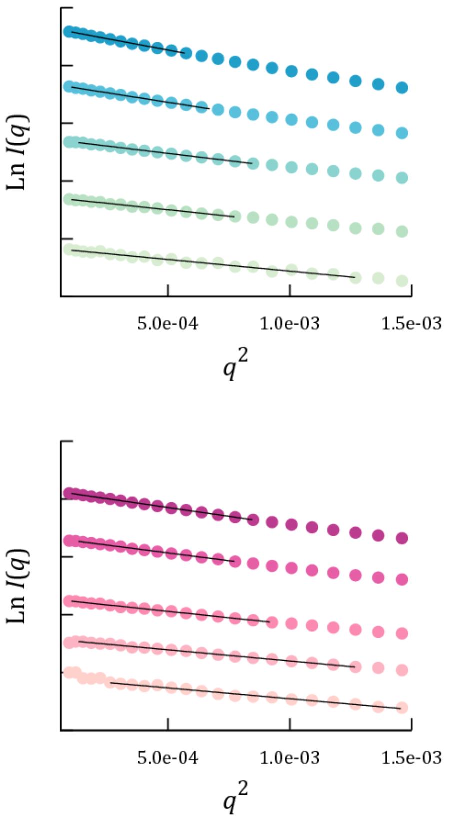

S3 Fig. Guinier analysis of SAXS data. Guinier analysis of SAXS data for $WzzB_{SF}$, top panel; and $WzzB_{SF}^{A107P}$, bottom panel. The first 25 points of each dataset are shown transformed as q^2 vs ln $I(q)$. Linear regressions used for determination of R_g and $I(0)$ are shown as black lines. Linearity in the fitted region is apparent for all datasets. These regions were determined using AUTORG. Larger ranges of points are selected for lower concentrations as selection is constrained to points where $q.R_g < 1.3$, and R_g increases with concentration for these samples.
